# Chromatin-based epigenetics of adult subventricular zone neural stem cells

**DOI:** 10.3389/fgene.2013.00194

**Published:** 2013-10-08

**Authors:** Gabriel Gonzales-Roybal, Daniel A. Lim

**Affiliations:** ^1^Department of Neurological Surgery, University of California at San FranciscoSan Francisco, CA, USA; ^2^Eli and Edythe Broad Center of Regeneration Medicine and Stem Cell Research, University of California at San FranciscoSan Francisco, CA, USA; ^3^Veterans Affairs Medical Center, University of California at San FranciscoSan Francisco, CA, USA

**Keywords:** epigenetics, chromatin modifications, neurogenesis, subventricular zone, neural stem cell, gene expression regulation

## Abstract

In specific regions of the adult mammalian brain, neural stem cells (NSCs) generate new neurons throughout life. Emerging evidence indicate that chromatin-based transcriptional regulation is a key epigenetic mechanism for the life-long function of adult NSCs. In the adult mouse brain, NSCs in the subventricular zone (SVZ) retain the ability to produce both neurons and glia for the life of the animal. In this review, we discuss the origin and function of SVZ NSCs as they relate to key epigenetic concepts of development and potential underlying mechanism of chromatin-based transcriptional regulation. A central point of discussion is how SVZ NSCs – which possess many characteristics of mature, non-neurogenic astrocytes – maintain a “youthful” ability to produce both neuronal and glial lineages. In addition to reviewing data regarding the function of chromatin-modifying factors in SVZ neurogenesis, we incorporate our growing understanding that long non-coding RNAs serve as an important element to chromatin-based transcriptional regulation, including that of SVZ NSCs. Discoveries regarding the epigenetic mechanisms of adult SVZ NSCs may provide key insights into fundamental principles of adult stem cell biology as well as the more complex and dynamic developmental environment of the embryonic brain.

## INTRODUCTION

While the vast majority of neurons in the adult mammalian brain arise during embryonic development, in specific brain regions, new neurons are born continuously throughout life. Under normal physiological conditions, adult neurogenesis persists in two distinct brain regions – the subgranular zone of the hippocampal dentate gyrus, and the subventricular zone (SVZ) of the lateral ventricles (Ming and Song, 2011; Fuentealba et al., 2012). These adult brain germinal zones harbor neural precursors – often referred to as neural stem cells (NSCs) – that proliferate and give rise to specific types of neurons. Adult NSCs have been identified in brains of rodents, cats, dogs, and primates, including that of humans (Lim et al., 2008). In the adult mouse, SVZ NSCs generate several subtypes of interneurons for the olfactory bulb (OB) as well as astrocytes and oligodendrocytes, the major glial cell types of the central nervous system (CNS).

Intriguingly, the adult NSCs that sustain life-long neurogenesis have many characteristics of mature astrocytes, a glial cell population known mostly for their supportive functions, which include the production of trophic factors, modulation of neuronal synapses, and maintenance of the blood–brain barrier (Molofsky et al., 2012). Astrocytes throughout the brain arise from radial glia, the primary embryonic neural precursor cell population that generates both neuronal and glial lineages during brain development (Kriegstein and Alvarez-Buylla, 2009). While most astrocytes descendent of radial glia become lineage restricted, dividing symmetrically to dramatically expand the population of astrocytes in the early postnatal brain (Ge et al., 2012), SVZ NSCs – which also arise from radial glia (Merkle et al., 2004) – retain the ability to produce both neurons and glia for the life of the animal. Thus, on a conceptual level, SVZ NSCs appear to have “preserved” some of the multi-lineage differentiation potential of their radial glial cell origin, whereas astrocytes that populate non-neurogenic brain regions seemingly “lose” their stem cell character. How adult NSCs maintain their neurogenic developmental potential is not well understood, but evidence indicates that both cell extrinsic developmental signals and cell-intrinsic epigenetic mechanisms are required (Ming and Song, 2011; Fuentealba et al., 2012; Hwang et al., 2012).

Epigenetics is the study of biological mechanisms that orchestrate a specific and heritable pattern of genome function without alterations to the DNA sequence. As with other multi-potent adult stem cell populations, SVZ NSCs must durably maintain an epigenetic state that enables both self-renewal and the production of multiple, differentiated cell types. This stem cell epigenetic state is likely to involve (1) the active expression of specific genes, (2) the repression of certain other loci, and (3) the maintenance of genes required for lineage specification (Vastenhouw et al., 2010). How is the expression of distinct sets of genes maintained in such “on,” “off,” and “poised” states? It is now recognized that the structure and function of chromatin – the complex of genomic DNA with histone proteins – comprises an important dimension to the transcriptional regulation that underlies cellular epigenetics. DNA methylation, histone post-translational modifications, and certain classes of non-coding RNAs (ncRNAs) can all strongly influence gene expression through their effect on chromatin structure, and it is becoming increasingly clear that these mechanisms play important roles in both development and the function of mature cell types. Interestingly, it is also now apparent that these molecular mechanisms interact and converge upon chromatin to exert transcriptional control and thus together may contribute to the epigenetic basis of cellular identity. The chromatin-based regulation of NSCs and their daughter cell lineages is a growing area of scientific investigation (Hsieh and Gage, 2004; Hirabayashi and Gotoh, 2010; Lee and Lee, 2010), and research into this aspect of the epigenetic landscape promises to reveal new, fundamental principles of neural development.

**FIGURE 1:**
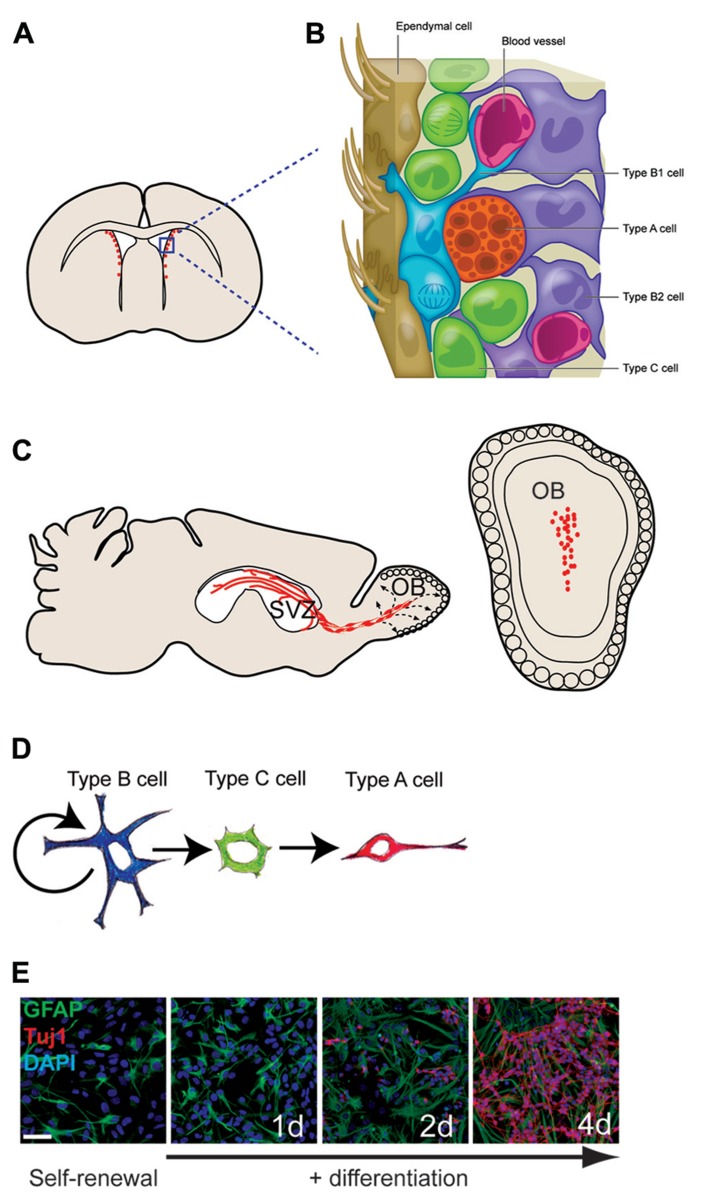
**The adult mouse SVZ: a tractable system for molecular-genetic studies of NSC differentiation.** **(A)** Coronal view of SVZ. **(B)** Enlarged view of SVZ: type B cells (blue) can contact the ventricle with a thin process extended between ependymal cells (brown); type C cells (green) are transit-amplifying cells that give rise to migratory type A cells (red). **(C)** Sagittal view showing paths of migratory neuroblasts (red, type A cells) to the OB. **(D)** The neuronal lineage of self-renewing type B cells. **(E)** SVZ NSC culture in self-renewal (left) and differentiation (right) conditions. The astrocyte marker GFAP in green, the neuronal marker Tuj1 in red, and DAPI in blue.

In contrast to the embryonic brain – where in multi-potent neural precursors are inherently transient, continually changing their developmental potential and location over time and with organ morphogenesis – in the adult SVZ, NSCs are developmentally stable and harbored in a well-defined cellular niche. SVZ NSCs can be efficiently cultured for molecular and biochemical studies, and when grown as monolayers, these cells recapitulate neurogenesis *in vitro* (**Figure 1E**) and generate OB interneurons when transplanted back to the SVZ *in vivo* (Scheffler et al., 2005; Merkle et al., 2007; Lim et al., 2009). The well-characterized, relatively simple developmental lineages of the adult SVZ, and the availability of a relevant NSC culture system, make this germinal zone particularly tractable for molecular-genetic studies of NSCs and their differentiation. Such integrated *in vivo* and *in vitro* SVZ studies have been used to elucidate key developmental principles regarding the role of signaling molecules, transcription factors, microRNAs, and – more recently – chromatin modifiers and long non-coding RNAs (lncRNAs; Ihrie et al., 2011; Fuentealba et al., 2012; Hwang et al., 2012).

In this review, we first examine the adult SVZ NSCs from a conceptual epigenetic perspective, surveying those aspects of SVZ NSC development and function that appear to involve cell-intrinsic epigenetic mechanisms. To next discuss the biology of adult SVZ NSCs in the context of chromatin-based transcriptional regulation, we provide a brief overview of relevant molecular mechanisms. For more comprehensive review of epigenetic mechanisms, the identity and mechanisms of major chromatin regulators, and new hypotheses regarding the function of chromatin modifications in transcriptional regulation, we refer the reader to a series of excellent reviews (Kouzarides, 2007; Bannister and Kouzarides, 2011; Badeaux and Shi, 2013; Bergman and Cedar, 2013; Calo and Wysocka, 2013). Here, we center our review and discussion upon those chromatin-modifiers that have been shown to have important roles in SVZ neurogenesis. We further incorporate recent studies of lncRNAs that demonstrate their role in the targeting and function of chromatin-modifying complexes, and highlight how a recent study of lncRNA expression and function in the SVZ provides new inroads for the discovery of chromatin-based transcriptional regulation in cellular development. Discovering the fundamental biological mechanisms that enable long-term SVZ NSC function may provide important insight into broader molecular principles that govern adult stem cell function in other tissues, and, further, may aid our understanding of how complex gene expression patterns are established and maintained in the much more complex and dynamic environment of the developing brain.

### THE ADULT MOUSE SVZ: A GERMINAL ZONE WITH ENDURING NEUROGENESIS AND GLIOGENIC POTENTIAL

The mammalian brain contains fluid-filled spaces called ventricles, and in adult mice, several thousand NSCs are distributed along the lateral ventricle walls (**Figures 1A,B**). These NSCs reside directly adjacent to the ventricle in a region that has been loosely termed the SVZ. Throughout life, these SVZ NSCs produce neuroblasts that migrate anteriorly to the OB where they differentiate into neurons and integrate into local circuits (Petreanu and Alvarez-Buylla, 2002; Carleton et al., 2003; **Figure 1C**). Several distinct interneuron subtypes are generated by the SVZ (Whitman and Greer, 2009), and, as we later discuss, these discrete neuronal lineages appear to be determined by cell-intrinsic epigenetic factors. Estimates indicate that thousands of new OB neurons are generated every day (Lois and Alvarez-Buylla, 1994), and this continuous adult neurogenesis contributes importantly to the neuro-physiological processing of olfactory information. SVZ NSCs also produce oligodendrocytes – the glial cell type that insulates neuronal axons with a myelin sheath – in both normal *in vivo* conditions and in response to demyelinating lesions (Picard-Riera et al., 2002; Menn et al., 2006). Brain injury also induces the SVZ to produce astrocytes that migrate to the injury site (Benner et al., 2013). Thus, the SVZ retains the ability to generate both neurons and glia long after birth of the animal.

While it is still unclear whether SVZ NSCs can self-renew indefinitely *in vivo*, and the rate of neurogenesis does indeed decrease with age, robust levels of SVZ neurogenesis persist for the life of the adult mouse (Tropepe et al., 1997; Jin et al., 2003; Maslov et al., 2004; Shook et al., 2012). Furthermore, NSCs have been identified in the SVZ of very old mice, and such multi-potent precursors can be isolated and propagated in culture. Thus, at least some SVZ NSCs preserve the cellular “competence” to generate neurons, astrocytes, and oligodendrocytes for upward of 2 years after birth.

### THE UNIQUE GLIAL CELL IDENTITY OF SVZ NSCs

While stem cells are traditionally thought of as being phenotypically immature and undifferentiated, adult mouse SVZ NSCs – called type B1 cells – have many characteristics of mature brain astrocytes, a glial cell type known to have various support functions for the brain. Type B1 cells express a number of glial markers including the glial-fibrillary acidic protein (GFAP; Doetsch et al., 1997), glutamate aspartate transporter (GLAST), and brain lipid binding protein (BLBP). Furthermore, type B1 cells have endfeet on blood vessels, which is typical of astrocytes found in the gray matter of the brain (Mirzadeh et al., 2008; Shen et al., 2008; Tavazoie et al., 2008).

Type B1 cells give rise to transit amplifying precursors (type C cells), which generate neuroblasts (type A cells) that migrate to the OB (Doetsch et al., 1999). Based on *in vitro* studies, it is believed that type B1 cells undergo asymmetric division for self-renewal and the production of type C cells. *In vivo* analysis indicates that type C cells then divide symmetrically approximately three times before becoming type A cells, which divide one or two more times while en route to the OB (Ponti et al., 2013). Type A cells migrate by sliding along one another within elongated cellular aggregates called chains (Lois et al., 1996; Wichterle et al., 1997). This chain migration of type A cells occurs within a network of interconnecting paths that coalesce into the rostral migratory stream (RMS) at the anterior SVZ (Doetsch and Alvarez-Buylla, 1996). The RMS carries the neuroblasts into the OB where they then differentiate into interneurons that become incorporated into local circuits (Petreanu and Alvarez-Buylla, 2002; Carleton et al., 2003; see **Figure 1** for details of SVZ architecture; **Figure 1C**)

Most type B1 cells make direct contact with the brain ventricle via specialized apical processes. These apical processes project between the ependymal cells that line the luminal surface of the brain ventricles. When viewed *en face*, the thin apical endings of type B1 cells cluster together at the center of a rosette of ependymal in a repeated “pinwheel” pattern (Mirzadeh et al., 2008). Whereas ependymal cells bear several motile cilia that move cerebrospinal fluid through the brain ventricles, the apical surface of each type B1 cell has a single, non-motile cilium lacking the central pair of microtubules (Doetsch et al., 1999). Cilium with this 9 + 0 microtubule arrangement are called the primary cilium and is present on embryonic neuroepithelial cells (Sotelo and Trujillo-Cenoz, 1958; Stensaas and Stensaas, 1968), adult avian brain neuronal precursors (Garcia-Verdugo et al., 1998), and NSCs of the adult hippocampus (Han et al., 2008). In some cells, primary cilium is required for sonic hedgehog (Shh) signaling, and this cellular structure may have additional roles in the transduction of other signaling molecules. While the role of primary cilium in type B1 cells is not yet known, its presence is one of the defining morphological characteristics of the SVZ NSCs.

The glial cell identity of adult SVZ NSCs is particularly intriguing when examined in the broader scope of embryonic brain development. It is now well established that a specialized type of glia – radial glia – gives rise to both neurons and glia found in essentially all regions of the adult brain (Noctor et al., 2001; Campbell and Gotz, 2002; Anthony et al., 2004). Radial glial cells are thus responsible for generating a tremendous variety of cell types. As we discuss in sections below, their developmental potential relates to both their temporal and regional identity. At early postnatal ages, ventral brain radial glia give rise to SVZ NSCs (Merkle et al., 2004). Interestingly, type B1 cells share certain morphological and spatial characteristics of embryonic radial glia. Do type B1 cells epigenetically “preserve” the neurogenic potential of their radial glial origin? What epigenetic mechanisms are required for the establishment and maintenance of adult NSCs? We next review aspects of radial glial biology that support, on a conceptual level, the notion that cell-intrinsic epigenetic mechanisms play a critical role in the development and long-term neurogenic function of SVZ type B1 cells.

## RADIAL GLIA: PRECURSOR CELLS OF BOTH NEURONS AND GLIA

Radial glial cells are a non-neuronal cell type that arises from the early embryonic neuroepithelium. These cells are so-named for their characteristic radial morphology and glial cell properties. The cell bodies of radial glia reside in the ventricular zone (VZ), and – like SVZ type B1 cells – maintain apical contact with the adjacent brain ventricle. Unlike adult SVZ NPCs, however, radial glia extend a thin radial process from their soma to the pial surface of the developing brain. Throughout development, radial glia maintain this apical–basal contact: as the brain grows with the progressive addition of newly born cells, radial glial cells elongate their basal process, and both apical and basal contacts are preserved (Kriegstein and Alvarez-Buylla, 2009).

In the mouse, neuroepithelial cells begin transforming into radial glia at around embryonic day 9 (E9). Shortly after this time, these cells begin expressing glial markers including GLAST, BLBP, and, in some species, GFAP (Hartfuss et al., 2001; Mori et al., 2005). Furthermore, radial glial cells express glycogen granules, which are found in astroglia but not neuroepithelial cells.

Multiple cell-fate analyses demonstrate that radial glia – when considered as a population – give rise to neurons and glia in essentially all parts of the brain. However, this is not to say that each individual radial glial cell is cell-intrinsically capable of giving rise to the entire diversity of cell types found in the adult brain. For instance, radial glia in the dorsal regions generate different types of neurons than those in the ventral brain. Furthermore, the developmental potential of embryonic neural precursors also changes over time. That is, early embryonic radial glia generate different neural cell types than those later in development. Interestingly, as we next discuss, it appears that this “progressive” change in developmental potential is in part cell-intrinsic and epigenetically stable.

### EVIDENCE FOR A TIME-DEPENDENT RESTRICTION TO THE DEVELOPMENTAL POTENTIAL OF EMBRYONIC NEURAL PRECURSORS

The mammalian cortex has a laminar structure, and the development of the six layers of major projection neurons proceeds in an “inside out” manner: neurons of the deepest layer are born first, and the more superficial neurons are born later in a sequential fashion, with the outer-most neurons born last (Molyneaux et al., 2007). Interestingly, the developmental potential of embryonic neural precursors becomes restricted by later stages of corticogenesis: cells isolated from the VZ of older embryos (when superficial neurons are being generated) only give rise to outer layer neurons even when transplanted to the younger embryonic brain (when deep layer neurons are being born; Frantz and McConnell, 1996). Thus, the “clockwork” of cortical neurogenesis appears to be predominantly cell-intrinsic. Furthermore, this time-dependent restriction of neurogenic potential is observed in precursors that have undergone mitosis in the younger recipient brain, indicating that the temporal identity of these embryonic neural precursors is epigenetically stable (Desai and McConnell, 2000).

The production of most glia follows embryonic neurogenesis. Toward the end of brain development, radial glia begin to produce astrocytes. Around this time, most radial glia lose their apical contact and translocate their soma away from the ventricle. This transformation of radial glial cells into astrocytes has been directly observed with time-lapse microscopy, and *in vivo* cell fate analysis support this lineage relationship (Noctor et al., 2008).

Both the sequential production of neuronal subtypes and the timing of when embryonic neural progenitors switch from neurogenesis to gliogenesis are also observed in cell culture studies. Single cortical precursor cells isolated from the E10–11 mouse brain produce neurons until gliogenesis initiates 10 days later, similar to the kinetics of the developmental transition observed *in vivo* (Qian et al., 2000). Furthermore, cortical neural precursors derived from embryonic stem cells (ESCs) maintain the “clockwork” of cortical neuron subtype production in addition to the neurogenic to gliogenic switch (Gaspard et al., 2008). These cell culture studies further support the notion that cell-intrinsic epigenetic mechanisms underlie the progressive change in developmental potential of neural precursor cells.

## ASTROCYTES IN NON-NEUROGENIC BRAIN REGIONS: CONTINUED PROGRESSIVE RESTRICTION OF DEVELOPMENTAL POTENTIAL?

The number of astrocytes increases dramatically in the first three postnatal weeks in mice. In the cortex – a non-neurogenic region in the postnatal and adult mouse – this glial cell expansion results from the symmetrical division of local astrocytes (Ge et al., 2012). These proliferative, GFAP and BLBP-positive cells have morphologies and electrophysiological properties of maturing astrocytes. These cortical astrocytes no longer express Nestin, an intermediate filament protein enriched in radial glia, suggesting a progressive “loss” of neural precursor cell identity. Do “young” cortical astrocytes – those that have recently arisen from radial glial cells – transiently retain an epigenetic state permissive for neurogenesis?

Two lines of evidence support this notion. First, when isolated from early postnatal mouse cortex, GFAP-expressing cells can behave as NSCs *in vitro*, proliferating to form clusters of cells called neurospheres that are capable of differentiating into all three major neural and glial lineages. However, in mice 14 days and older, these GFAP-positive cells no longer generate multi-potent neurospheres. These data suggest that “young” astrocytes represent a transition state that has not yet “locked down” a glial, non-neurogenic identity and thus is still capable of generating neuronal lineages given the proper extrinsic signals (Laywell et al., 2000).

Secondly, early postnatal astrocytes can be transdifferentiated into neurons with the overexpression of neurogenic transcription factors. For instance, overexpression of *Dlx2* – a transcription factor with key roles in adult SVZ and embryonic interneuron development – can transform postnatal day 7–9 (P7–9) cortical astrocytes into functional inhibitory interneurons (Heinrich et al., 2010). Like neurosphere formation, this neurogenic “competence” of cortical astrocytes appears to be limited to early postnatal mice; when isolated from older mice, cortical astrocytes do not differentiate into neurons with transcription factor overexpression. Taken together, these experiments suggest that the developmental potential of astrocytes in non-neurogenic regions becomes progressively restricted.

Can fully mature astrocytes return to a “younger” state permissive for self-renewal and multi-lineage differentiation? In response to trauma, astrocytes in the injured area re-express Nestin (Pekny and Nilsson, 2005) and can generate multi-potent neurospheres. Furthermore, reactive astrocytes can be transdifferentiated into neurons via neurogenic transcription factor overexpression (Berninger et al., 2007). Thus, it appears that injury can induce adult reactive astrocytes to regain some of the neurogenic competence similar to that of glia in the early postnatal brain (Sirko et al., 2013).

## SVZ TYPE B1 CELLS: MAINTENANCE OF A RADIAL GLIAL-LIKE IDENTITY?

During early postnatal development, radial glial cells give rise to SVZ type B1 cells. Unlike cortical astrocytes, which lose their apical contact with the ventricle, type B1 cells maintain this morphological and spatial characteristic (Mirzadeh et al., 2008). Neurospheres and other types of NSC cultures can be established from SVZ cells throughout life, indicating the enduring NSC character of cells in this brain region. While extrinsic factors are implicated in the maintenance of adult neurogenesis, the SVZ “niche” alone does not appear to be sufficient. What are the cell-intrinsic mechanisms that help maintain the neurogenic developmental potential of type B1 cells?

## CHROMATIN-BASED TRANSCRIPTIONAL REGULATION

In the nucleus, genomic DNA is condensed by chromosomal proteins into a dynamic polymer called chromatin. The local structure of chromatin can influence gene expression, and some chromatin modifications are heritable through cell divisions. Thus, changes to chromatin structure can engage and maintain particular genetic programs and likely play a critical role in both stem cell maintenance and daughter cell differentiation.

The basic subunit of chromatin is the nucleosome, which is comprised of 146 bp of DNA wrapped approximately twice around an octamer of the four core histone proteins (H3, H4, H2A, and H2B). Histone variants also exist and likely play a role in the epigenetics of transcriptional regulation, but we do not include these in our discussion, as we are not aware of data regarding their function in adult SVZ neurogenesis.

There are two basic mechanisms for modifying chromatin states: (1) non-covalent modifications of protein–protein and protein–DNA interactions including ATP-dependent chromatin remodeling, and (2) covalent changes to chromatin such as DNA methylation and histone amino acid modifications. Core histones are subject to over 100 different post-translational modifications, and while most are still poorly understood, it is clear that some of these covalent changes strongly correlate with gene expression. For instance, histone acetylation is generally associated with local transcription. Histone methylation can be associated with either transcriptional activation or repression, depending in part on which particular residue is modified. In a combinatorial fashion, it is likely that such covalent modifications comprise a “histone code” that determines local chromatin structure and thus epigenetically imposes specific transcriptional programs (Jenuwein and Allis, 2001).

As with our neural developmental discourse above, a central concept to our mechanistic discussion below relates to the observation that while most radial glia progressively lose their neurogenic potential, SVZ type B1 cells retain the ability to produce neurons throughout life. For this review, we next discuss select chromatin-based mechanisms that have been shown to regulate adult SVZ neurogenesis.

### HISTONE ACETYLATION: REQUIRED FOR EFFICIENT SVZ NEUROGENESIS

Histone acetylation – the first described histone modification – is associated with active transcription (Davie and Hendzel, 1994). Many lysine residues in the N-terminal tails of the core histone proteins can become acetylated by a number of different histone acetyltransferases (HATs). Histone lysine acetylation may facilitate transcription by “loosening” the intra- and inter-nucleosomal charge interactions by increasing the net negative charge of histones and repelling these proteins from DNA, thus increasing local DNA accessibility to the transcriptional machinery, including transcription factors. Additionally, acetylated histone lysine residues serve as a target for certain bromodomain-containing chromatin-remodeling factors (Kouzarides, 2007), which can also increase DNA accessibility through ATP-dependent nucleosome repositioning. CNS-specific deletion of either CBP or p300 HATs results in defective neural tube closure, indicating that these chromatin-modifying factors are critical at the earliest stages of brain development (Yao et al., 1998; Tanaka et al., 2000).

Histone deacetylation is associated with transcriptional silencing, and this covalent change is catalyzed by histone deacetylases (HDACs), of which there are four classes: class I (HDAC1, 2, 3, and 8), class II (HDAC4, 5, 6, 7, 9, and 10), class III (Sirt1–7), and class IV (HDAC11).

Subventricular zone neurosphere cultures treated with class I and II HDAC inhibitors exhibit increased neuronal production and decreased oligodendrocyte differentiation, suggesting a role for this chromatin modification in neural fate determination (Siebzehnrubl et al., 2007). Class I and II HDACs also have been implicated in SVZ NSC self-renewal, as inhibitors to these enzymes increase the expression of cell cycle inhibitors, correlating with a block in G1-S phase progression (Zhou et al., 2011). Furthermore, treatment of postnatal mice with class I/II HDAC inhibitors strongly perturbs SVZ neurogenesis (Foti et al., 2013).

In the postnatal SVZ, HDAC1 is expressed in GFAP-positive cells – presumably including type B1 cells – and HDAC2 is expressed in the migratory type A cells (MacDonald and Roskams, 2008; Montgomery et al., 2009; Foti et al., 2013). It appears that HDAC2 is also expressed in type C cells of the adult SVZ, suggesting a role for this enzyme in neuronal differentiation, and HDAC2 deletion targeted to SVZ NSCs results in defective production of OB (Jawerka et al., 2010).

Histone deacetylases are known to interact with transcription factors and can be targeted to specific loci. Tailless (TLX) is a transcription factor expressed in SVZ type B1 cells and is required for NSC self-renewal and adult neurogenesis (Sun et al., 2007). TLX interacts with HDAC3 and HDAC5, and in adult NSCs, TLX recruits these HDACs to cell cycle inhibitor p21Cip1/WAF1 and the *Pten* tumor suppressor, leading to their transcriptional repression. These data support a model in which TLX regulates SVZ NSC function in part through chromatin-based mechanisms. More generally, the above studies indicate that histone acetylation is a critical component of chromatin-based transcriptional regulation required for SVZ neurogenesis.

## DNA METHYLATION: A PLAYER IN THE NEUROGENIC–GLIOGENIC SWITCH OF EMBRYONIC DEVELOPMENT

DNA methyltransferases (DNMTs) mediate the covalent addition of methyl groups to the C5 position of cytosine residues. In mammalian cells, this DNA methylation typically occurs within CpG dinucleotides. In promoter regions, DNA methylation generally correlates with transcriptional repression. DNMT1 is constitutively expressed in dividing cells, and during DNA replication, this enzyme symmetrically propagates methylation to the nascent daughter strand. While DNMT1 appears to serve primarily to maintain DNA methyl marks, DNMT3A and DNMT3B are de novo methyltransferases, capable to catalyzing cytosine methylation at new genomic locations (reviewed in Law and Jacobsen, 2010).

The timing of the neurogenic-gliogenic “switch” during development appears to be in part regulated by DNA methylation. The proximal promoter of astrocytic genes *GFAP* and *S100β* contain response elements to the signal transducer and activator of transcription (STAT) transcription factor; during the neurogenic phase of embryonic development, CpG sites in these response elements are methylated, which blocks STAT binding (He et al., 2005). Indeed, even overexpression of STAT3 in early embryonic NPCs does not increase the expression of these astrocytic genes (Bonni et al., 1997; He et al., 2005). As development progresses, methylation of STAT binding elements in astrocytic genes decreases and STAT3 becomes enriched at these sites, correlating with astrocyte differentiation. Furthermore, ablation of *Dnmt1* results in premature production of astrocytes, suggesting that the loss of DNA methylation in part releases the restriction of astrocyte production during early neurogenic phases of development.

How DNA methylation at the astrocytic STAT binding elements is lost during gliogenic phases is not known, however, recent discoveries raise the potential role of active demethylation. Ten–eleven translocation 1–3 (TET 1–3) proteins are a family of enzymes that can convert 5-methylcytosine to 5-hydroxymethylcytosine (5hmC), which may be an important intermediate to DNA demethylation via base-excision repair. Recently, Zhang et al. (2013) demonstrated that mice lacking *Tet1* have impaired adult hippocampal neurogenesis; in *Tet1*-deficient hippocampal NPCs, a set of genes related to progenitor cell proliferation are hypermethylated and exhibit decreased expression. Future work may reveal roles for TET proteins in the plasticity of cell identity and developmental potential.

## NON-CANONICAL ROLE OF DNA METHYLATION IN SVZ NEUROGENESIS

While DNA methylation is well known for its repressive role at gene promoters, DNA methylation is also abundant within many gene bodies. While the role of non-promoter DNA methylation still remains unclear, in the adult SVZ, DNMT3A-dependent non-promoter DNA methylation appears to facilitate the transcription of key neurogenic genes (Wu et al., 2010). Mice null for *Dnmt3a* have severely diminished SVZ neurogenesis, resulting in smaller OBs. Genome-wide analysis of DNA methylation and other histone modifications suggested a novel mechanism of activation, that Dnmt3a methylates DNA proximal to the promoters of neurogenic genes like *Dlx2*, which antagonizes the placement of repressive histone modifications. These data suggest that SVZ type B1 cells require *Dnmt3a* to help maintain epigenetic activation of specific loci. Interestingly, *Dnmt3a* is not expressed in non-neurogenic brain regions, suggesting that this de novo DNA methyltransferase is a key component to the maintenance of adult neurogenesis.

## POLYCOMB GROUP AND TRITHORAX GROUP CHROMATIN-MODIFYING FACTORS: CRITICAL REGULATORS OF ADULT SVZ NEUROGENESIS

The polycomb group (PcG) and trithorax group (trxG) gene products, originally described in *Drosophila*, repress or activate transcription, respectively, to control the specification and maintenance of cell identity. By assembling into large multi-protein complexes that modify chromatin structure, they organize the genome regionally into transcriptionally silent or active states.

Polycomb group complexes silence gene expression by a number of different mechanisms including the covalent modification of histones by ubiquitination and methylation. Of all of the histone modifications mediated by PcG complexes, histone 3 lysine 27 trimethylation (H3K27me3) is the best understood. Polycomb repressive complex 2 (PRC2) contains EZH2, a catalytically active core component that trimethylates H3K27. Bmi1 and Ring1b are components of the polycomb repressive complex 1 (PRC1) that recognizes H3K27me3 and participates in transcriptional silencing through multiple mechanisms, including chromatin compaction. In embryonic brain development, members of both PRC1 and PRC2 are necessary for regulating the transition from neurogenesis to gliogenesis, suggesting that chromatin-based mechanisms play a key role in establishing the developmental potential of neural precursor cells (Fasano et al., 2009; Pereira et al., 2010).

In the adult SVZ, polycomb-mediated gene silencing is required for the maintenance of the NSC population. With increasing age, *Bmi1*-deficient mouse cells generate fewer SVZ neurospheres as compared to wildtype control (Molofsky et al., 2003). Conversely, overexpression of *Bmi1* rescues this age-dependent decline in neurosphere formation and dramatically increases SVZ cell proliferation *in vivo*, suggesting a role for Bmi1 in the self-renewal of SVZ NSCs (Fasano et al., 2009; He et al., 2009). Furthermore, the defect in neurosphere production from BMI1 knockout mice is rescued by the null mutations of the cell cycle inhibitors p16, p19, and p21, suggesting that the repression of cell cycle regulators is a critical aspect of *Bmi1*-dependent adult neurogenesis.

Opposing the effects of PcG complexes, trxG complexes activate gene expression. *Mixed lineage leukemia-1* (*Mll1*) is the prototypic trxG homolog of the mixed lineage leukemia (MLL) family. There are several MLL family members, and these appear to have independent roles in development. In addition to non-covalent chromatin remodeling processes, MLL proteins can catalyze histone 3 lysine 4 trimethylation (H3K4me3) – a mark that is permissive for transcription – and recruit H3K27me3-specific demethylases (Dou et al., 2006; De Santa et al., 2007; Swigut and Wysocka, 2007).

*Mll1* is expressed in the SVZ lineage, and deletion of *Mll1* targeted to SVZ NSCs results in a severe reduction in neurogenesis, but not gliogenesis (Lim et al., 2006). In SVZ monolayer cultures, *Mll1*-deleted cells proliferate normally and maintain the expression of NSC markers such as Nestin and Sox2. However, during differentiation, Mll1-deleted SVZ cells are unable to properly induce expression of the neurogenic transcription factor, DLX2. Enforced expression of *Dlx2* in *Mll1*-deleted SVZ NSCs in culture could partially rescue neuronal differentiation, indicating that DLX2 is a key component of MLL1-dependent neurogenesis. The *Dlx2* locus is a direct target of MLL1, and, interestingly, H3K4me3 is not decreased at the *Dlx2* transcriptional start site (TSS) in *Mll1*-deleted cells, suggesting that other trxG family members can compensate for loss of MLL1. However, in the absence of MLL1, the *Dlx2* locus is enriched for repressive H3K27me3, correlating with the failure of proper *Dlx2* upregulation. MLL family members can physically interact with and recruit H3K27-specific demethylases UTX and JMDJ3 to specific loci. Thus, taken together, the data support a model in which MLL1 is required for the recruitment of H3K27-specific demethylase activity to specific neurogenic loci for neuronal differentiation.

While it is clear that PcG and trxG factors interact at specific genetic loci, how such targeting is achieved in mammalian cells is not well understood. The *Drosophila* genome harbors polycomb and trithorax response elements – specific sequences to which specific subunits of PcG and trxG bind – however, it does not appear that mammalian cells contain such genomic elements. A number of different mechanisms for PcG and trxG complex targeting in mammalian cells have been described including recruitment via transcription factor interactions and the localization to specific CpG islands. More recently, studies have emerged demonstrating the critical role of lncRNAs in the targeting of chromatin-modifying activities to specific loci.

## TARGETING PcG and trxG COMPLEXES: THE EMERGING ROLE OF LONG NON-CODING RNAs

The mammalian genome has been recently discovered to transcribe many thousands of lncRNAs – transcripts > 200 nucleotides long that have little evidence of protein coding potential – and a subset of these transcripts interacts with PcG and trxG chromatin-modifying factors to epigenetically regulate cellular fate (Khalil et al., 2009; Bertani et al., 2011; Wang et al., 2011). Before the advent of high-throughput sequencing technologies, only a handful of lncRNAs were known. The lncRNA Xist was discovered in the early 1990s, and an elegant combination of *in vivo* and *in vitro* studies established its critical role in X chromosome inactivation via polycomb-dependent mechanisms (reviewed in Plath et al., 2002). In the past decade, many more lncRNAs have been identified. Over 8000 human lncRNAs were recently annotated from RNA deep sequencing (RNA-seq) data, and similar catalogs have been generated from various mouse tissues and model organisms, leading to novel insights into their genomic structure and patterns of expression (Guttman et al., 2011; Ramos et al., 2013). For example, lncRNAs exhibit greater temporal and brain region specificity than the coding transcriptome, suggesting that these transcripts have cell-specific roles in both development and adult brain function.

Recent studies have demonstrated that many lncRNAs play key roles in cellular function and development. One emerging mechanistic theme is that lncRNAs can regulate gene expression through the targeting and recruitment of chromatin-modifying complexes. For instance, a specific portion of the Xist transcript binds PRC2 components and is required for efficient placement of H3K27me3 repressive marks upon the inactive X chromosome (Zhao et al., 2008). Many other lncRNAs are associated with PRC2 and play a role in transcriptional regulation. LncRNA *HOTAIR*, for example, interacts with PRC2 and is required for targeting PRC2 and H3K27me3 to specific genomic regions (Rinn et al., 2007). Intriguingly, *HOTAIR* also tethers PRC2 to other histone modifiers, forming a ribonucleoprotein complex that contains multiple enzymatic activities that confer a repressive chromatin state (Tsai et al., 2010). Furthermore, genome-wide analysis detects *HOTAIR* transcripts – even when overexpressed – at discrete genomic locations, suggesting that this lncRNA participates in the targeting of chromatin-modifying complexes.

**FIGURE 2:**
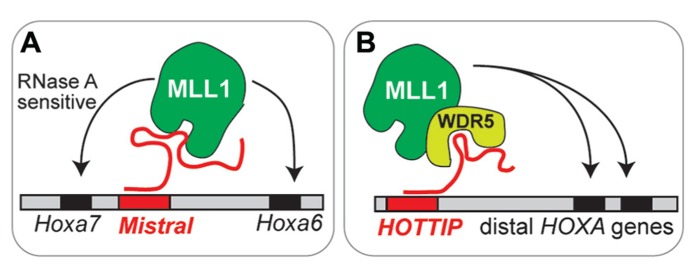
**Emerging models of lncRNA function in chromatin- modifying complexes.** **(A)** *Mistral* recruits MLL1 to specific *Hox* genes. This MLL1 binding is sensitive to RNase A, which degrades single stranded RNA. **(B)** *HOTTIP* recruits MLL1 through interaction with WRD5, a core component of MLL complexes.

Specific lncRNAs also play a role in the targeting of MLL1. *Mistral* binds MLL1 and targets it to *Hox* genes that are upregulated (Bertani et al., 2011). Similarly, lncRNA *HOTTIP* can target trxG-MLL1 complexes to upregulated *Hox* loci (Wang et al., 2011; **Figure 2**). Are lncRNA involved in the targeting of MLL1 to specific genomic regions (e.g., *Dlx2* transcriptional elements) in adult SVZ cells? At present, our understanding of lncRNA function in brain development is very limited: mice null for lncRNA *Evf2* have abnormal interneuron development and function (Bond et al., 2009), and morpholino inhibition of two different lncRNAs in zebrafish impairs brain development (Ulitsky et al., 2011). Do lncRNA play key roles in the epigenetic maintenance of adult neurogenesis?

### IDENTIFICATION OF lncRNAs RELATED TO ADULT SVZ NEUROGENESIS

To identify specific lncRNAs with potential roles in adult neurogenesis, we recently performed a comprehensive analysis of lncRNA expression in the SVZ NSC lineage (Ramos et al., 2013). First, we used Illumina-based RNA-seq and *ab initio* reconstruction of the transcriptome to generate an lncRNA catalog inclusive of adult NSCs and their daughter cell lineages. This lncRNA catalog informed a subsequent RNA capture-seq approach, which increased the read coverage and read length for our SVZ cell analysis, validating the transcript structure and expression of many novel lncRNAs. ChIP-seq analysis revealed that lncRNA loci – like key developmental genes – exhibit chromatin-based changes in a neural lineage-specific manner. Using custom lncRNA microarrays, we found that lncRNAs are dynamically regulated during neurogenesis *in vitro*. To define lncRNAs of the SVZ neurogenic lineage *in vivo*, we acutely isolated the major cell types of the SVZ with fluorescent activated cell sorting (FACS) for lncRNA expression analysis. Integration of these diverse experimental approaches stringently identified ~100 lncRNAs with potential roles in SVZ neurogenesis, including *Dlx1as*, a lncRNA encoded from an ultraconserved region of the *Dlx1/2* bigene cluster.

### Dlx1as: A lncRNA ENCODED FROM THE Dlx1/2 BIGENE CLUSTER

**FIGURE 3:**
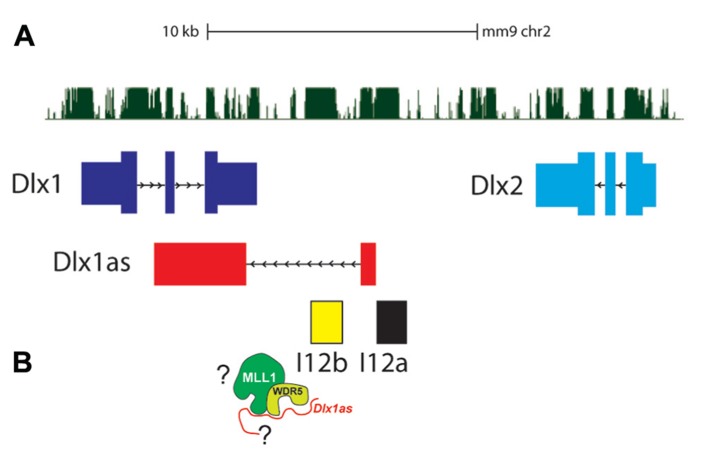
**The Dlx1/2 bigene cluster. (A)** Dlx1 (dark blue) and Dlx2 (light blue) are oriented in an inverted configuration separated by two enhancers, I12a (black) and I12b (yellow). The lncRNA Dlx1as transcript (red) consists of two “exons” that are spliced and polyadenylated. **(B)** Whether Dlx1as is a key component of MLL1 function at the Dlx1/2 locus is not known.

The *Dlx1/2* bigene cluster encodes two homeodomain-containing transcription factors, DLX1 and DLX2, which are critical neurogenic regulators in both embryonic brain development and adult SVZ neurogenesis (Ghanem et al., 2007; Petryniak et al., 2007; **Figure 3**). Mice null for *Dlx1/2* fail to develop GABAergic cortical and OB interneurons and do not survive; *Dlx1*-null mice survive to adulthood but suffer from epilepsy.

*Dlx1as* is transcribed from a TSS adjacent to the *I12a* enhancer, through the *I12b* enhancer, and the second exon overlaps *Dlx1* on the opposite strand (Ghanem et al., 2007; **Figure 3**). Interestingly, in autistic probands, single nucleotide polymorphisms (SNPs) have been found at the DLX1/2 locus, including the ultraconserved regions that correspond to *Dlx1as* (Liu et al., 2009).

shRNA-mediated *Dlx1as* knockdown reduces *Dlx1* and *Dlx2* in SVZ NSC monolayer cultures and strongly inhibits neurogenesis (Ramos et al., 2013). The mechanism by which *Dlx1as* regulates expression of *Dlx1* and *Dlx2* remain to be discovered. Furthermore, it is possible that *Dlx1as* is also required for neuronal differentiation independent of *Dlx1/2* regulation. However, based on the above mentioned findings describing a role for lncRNAs in targeting MLL complexes to *Hox* genes for positive transcriptional regulation, it is tempting to speculate that the localization of MLL1 to *Dlx1/2* regulatory elements requires *Dlx1as*, or other lncRNAs. Given that *Dlx* bigene clusters are genetically linked to *Hox* clusters (e.g., *Dlx1/2* is on the same chromosome of *HoxC* genes), and that *Dlx* and *Hox* genes appear to have co-evolved, perhaps the lncRNA-based mechanisms that regulate the function of polycomb and trithorax chromatin-modifying factors are also conserved among these evolutionarily conserved gene clusters (**Figure 3**).

## CONCLUDING REMARKS

### THE CHROMATIN-STATE OF THE Dlx1/2 BIGENE CLUSTER: A KEY ASPECT OF THE NEUROGENIC DEVELOPMENTAL POTENTIAL OF GLIAL CELLS?

The epigenetic state of multi-potent stem cells likely includes the maintenance of key developmental regulators in a transcriptionally silent but “poised” condition. In *Mll1*-deficient SVZ cells, the *Dlx2* promoter region is enriched for both H3K4me3 and H3K27me3 (Lim et al., 2009). Chromatin regions “bivalent” for both H3K4me3 and H3K27me3 are believed to play an important role in stem cell populations by maintaining developmental genes in a silenced state that is poised for activation or repression upon lineage commitment. While promoter region bivalency does appear to correlate with a poised transcriptional state, not all genes monovalent for H3K4me3 are transcriptionally active. In fact, in ESCs, approximately 28% of H3K4me3-monovalent genes are not expressed (Vastenhouw et al., 2010). Are there other epigenetic mechanisms that can maintain H3K4me3-monovalent genes in a transcriptionally poised state?

It appears that enhancers can be poised, too. Enhancers are DNA regulatory elements that drive lineage-specific gene expression, and emerging evidence indicate that enhancer activity is regulated by the local chromatin state. In particular, enrichment of histone 3 lysine 4 monomethylation (H3K4me1), H3K27me3, and binding of the p300 co-transcriptional activator is a signature of poised transcriptional enhancers. Replacement of H3K27me3 with H3K27 acetylation (H3K27ac) correlates with enhancer element activation. Thus, activation of poised enhancers may require the removal of H3K27me3 and the enzymatic addition of H3K27ac (Rada-Iglesias et al., 2011; Buecker and Wysocka, 2012). These recent observations suggest that chromatin-modifying factors – including PcG and trxG factors – play critical roles at both promoters and enhancers for the regulation of lineage-specific gene expression. Given that enhancer elements can drive cell-specific expression of reporter genes from basal promoters, the chromatin-state of enhancers may be a crucial – if not key – aspect of epigenetic cellular identity and developmental potential.

Activation of the *Dlx1/2* locus is required for adult SVZ neurogenesis from type B1 cells, which arise from ventral brain radial glia in early postnatal development. Expression of *Dlx1* and *Dlx2* is also required for the generation of specific interneuron populations from ventral brain radial glial cells during embryonic development. Thus, the *Dlx1/2* bigene cluster likely exists in a transcriptionally poised state in certain embryonic radial glia as well as adult type B1 cells. Although the nature of this epigenetic state is still under investigation, based on our discussions above, we may speculate that a specific chromatin signatures at the *Dlx1/2* locus in part underlies the neurogenic potential of glial cells in both the embryo and adult SVZ. Consistent with this notion, we may further hypothesize that neurogenic loci – including their regulatory regions – become progressively repressed at the level of chromatin in non-neurogenic, non-SVZ astrocytes. Is the poised state of *Dlx1/2* in SVZ type B1 cells related to chromatin modifications at the intragenic enhancers, promoter regions, or both? Which chromatin-modifying factors are required to establish and maintain this ability to rapidly activate the potent *Dlx1* and *Dlx2* neurogenic transcription factors? Are lncRNAs required for the local recruitment of PcG and trxG factors? By focusing in on the complex orchestration of chromatin-based modifications that occur at *Dlx1/2* in neurogenic glial cells through development and into adulthood, we may discover fundamental principles of the epigenetics that underlie both regional and temporal differences in developmental potential of neural precursors.

### THE COMPLEX INTERPLAY OF CHROMATIN MODIFICATIONS AND THEIR EFFECTORS

From our discussion above, it is apparent that the transcriptional regulation of key developmental regulators involves the complex interplay of multiple chromatin modifications and their effectors. For instance, based on our current mechanistic models, the maintenance of *Dlx1/2* in a transcriptionally poised state may involve polycomb factors at both enhancer and promoter regions. *Dlx1/2* expression requires trxG MLL1 protein and possibly the action of H3K27me3-specific demethylases, which have been implicated in enhancer and promoter activation. Additionally, the activity of HATs and/or inhibition of HDAC activity are also likely required at the *Dlx1/2* bigene cluster for efficient neurogenesis. The binding of PRC2 complexes to *Dlx2* non-promoter regions appears to be antagonized by DNMT3A-dependent de novo DNA methylation, indicating an interplay between histone and DNA modifications. Furthermore, polycomb and trithorax proteins must somehow become localized to *Dlx1/2* regulatory elements, and emerging data implicate lncRNAs in the targeting of chromatin-modifying complexes, including MLL1 and EZH2. Clearly, as we continue to explore the epigenetic landscape neural development, we will need to investigate in parallel the multitude of chromatin modifications. By experimentally testing models of the functional interactions between chromatin modifications and their effectors at critical genomic regions such as *Dlx1/2*, we may discover mechanistic themes relevant to the epigenetics of embryonic development as well as adult stem cell populations.

## Conflict of Interest Statement

The authors declare that the research was conducted in the absence of any commercial or financial relationships that could be construed as a potential conflict of interest.
